# Distinct responses of neurons and astrocytes to TDP-43 proteinopathy in amyotrophic lateral sclerosis

**DOI:** 10.1093/brain/awz419

**Published:** 2020-02-10

**Authors:** Phillip Smethurst, Emmanuel Risse, Giulia E Tyzack, Jamie S Mitchell, Doaa M Taha, Yun-Ru Chen, Jia Newcombe, John Collinge, Katie Sidle, Rickie Patani

**Affiliations:** 1 Department of Neuromuscular Disease, UCL Queen Square Institute of Neurology, Queen Square, London, WC1N 3BG, UK; 2 The Francis Crick Institute, 1 Midland Road, London NW1 1AT, UK; 3 MRC Prion Unit at UCL, UCL Institute of Prion Diseases, Courtauld Building, 33 Cleveland Street, London W1W 7FF; 4 Genomics Research Center, Academia Sinica, 128, Academia Road, Section 2, Nankang District, Taipei 115, Taiwan; 5 NeuroResource, Department of Neuroinflammation, UCL Queen Square Institute of Neurology, 1 Wakefield Street, London, WC1N 1PJ, UK

**Keywords:** motor neurons, astrocytes, TDP-43, aggregation, oligomers

## Abstract

Amyotrophic lateral sclerosis (ALS) is a fatal and incurable neurodegenerative disease caused by motor neuron loss, resulting in muscle wasting, paralysis and eventual death. A key pathological feature of ALS is cytoplasmically mislocalized and aggregated TDP-43 protein in >95% of cases, which is considered to have prion-like properties. Historical studies have predominantly focused on genetic forms of ALS, which represent ∼10% of cases, leaving the remaining 90% of sporadic ALS relatively understudied. Additionally, the role of astrocytes in ALS and their relationship with TDP-43 pathology is also not currently well understood. We have therefore used highly enriched human induced pluripotent stem cell (iPSC)-derived motor neurons and astrocytes to model early cell type-specific features of sporadic ALS. We first demonstrate seeded aggregation of TDP-43 by exposing human iPSC-derived motor neurons to serially passaged sporadic ALS post-mortem tissue (spALS) extracts. Next, we show that human iPSC-derived motor neurons are more vulnerable to TDP-43 aggregation and toxicity compared with their astrocyte counterparts. We demonstrate that these TDP-43 aggregates can more readily propagate from motor neurons into astrocytes in co-culture paradigms. We next found that astrocytes are neuroprotective to seeded aggregation within motor neurons by reducing (mislocalized) cytoplasmic TDP-43, TDP-43 aggregation and cell toxicity. Furthermore, we detected TDP-43 oligomers in these spALS spinal cord extracts, and as such demonstrated that highly purified recombinant TDP-43 oligomers can reproduce this observed cell-type specific toxicity, providing further support to a protein oligomer-mediated toxicity hypothesis in ALS. In summary, we have developed a human, clinically relevant, and cell-type specific modelling platform that recapitulates key aspects of sporadic ALS and uncovers both an initial neuroprotective role for astrocytes and the cell type-specific toxic effect of TDP-43 oligomers.

## Teaser

Smethurst *et al*. develop a human, clinically relevant modelling platform that recapitulates key aspects of sporadic ALS. They demonstrate cell type-specific vulnerability to both seeded aggregation (induced by post-mortem tissue extracts) and upon treatment with highly purified recombinant TDP-43 oligomers.

## Introduction

Amyotrophic lateral sclerosis (ALS) is caused by the selective degeneration of motor neurons in the brain and spinal cord, which results in rapidly progressive paralysis and invariable death, commonly by respiratory failure. ALS has a lifetime risk of ∼1 in 400 in individuals with European ancestry (Al-Chalabi and Hardiman, 2013). Ten per cent of cases are hereditary, termed familial ALS, while the majority are sporadic. Importantly, the majority of studies have focused on familial ALS, while experimental study of sporadic ALS has remained relatively inaccessible. However, induced pluripotent stem cell (iPSC) models now allow the generation of renewable human cell type-specific models for investigating disease mechanisms.

An increasing number of neurodegenerative diseases are considered to have ‘prion-like’ characteristics (Walker and Jucker, 2015), which include deposition of assemblies of misfolded proteins, their propagation and spread in the neuraxis. These may involve distinct strains of propagating seeds and neurotoxicity mediated via oligomeric species or more complex multichain assemblies of the pathological protein involved (Collinge and Clarke, 2007; Collinge, 2016). One of the principal drivers of such a prion-like proteinopathy is ‘templated seeding’ whereby misfolded protein assemblies propagate by addition of monomers to adopt the same misfolded form with subsequent fission of elongating assemblies to generate more seeds. A key pathological hallmark of ALS is cytoplasmically mislocalized and aggregated TDP-43 in motor neurons and glia within the brain and spinal cord of patients (Arai *et al.*, 2006; Neumann *et al.*, 2006).

Against this background, we and others have shown that TDP-43 pathology can be directly and reliably reproduced in HEK293 and NSC-34 cells via a templated seeding reaction from human sporadic ALS post-mortem brain and spinal cord tissue (Furukawa *et al.*, 2011; Tsuji *et al.*, 2012; Nonaka *et al.*, 2013; Fang *et al.*, 2014; Smethurst *et al.*, 2016). In addition, we showed that resulting aggregates morphologically resemble inclusions observed in ALS post-mortem tissue and can propagate from one cell to another in a prion-like fashion. However, much remains to be discovered about the mechanisms of TDP-43 toxicity and spread, and the underlying cell type-specific vulnerability to such phenomena. Indeed, there is accumulating evidence implicating astrocytes in ALS (Tyzack *et al.*, 2016; Yamanaka and Komine, 2018).

Here we have induced a pathological TDP-43 seeded aggregation reaction in control human iPSC-derived motor neurons and astrocytes on an endogenous background level of TDP-43 expression using sporadic ALS spinal cord tissue extract serially passaged in cell culture (spALS). These TDP-43 aggregates were phosphorylated and morphologically representative of ALS post-mortem tissue. We found that astrocytes could also be seeded with TDP-43 aggregates but at lower frequency than motor neurons, implying cell-type specific differences in vulnerability. We found that proteasomal inhibition could both exacerbate seeded TDP-43 aggregation and significantly increase cell death in motor neurons when compared to astrocytes. We were also able to demonstrate that TDP-43 pathology spreads from motor neurons to astrocytes preferentially, but could also be observed spreading from astrocytes to motor neurons. We next found that astrocytes—both by physical co-culture and conditioned medium experiments—are neuroprotective to seeded aggregation within motor neurons by reducing (mislocalized) cytoplasmic TDP-43, TDP-43 aggregation and cell toxicity. Furthermore, we detected TDP-43 oligomers in these spALS spinal cord extracts, and as such demonstrated that highly purified recombinant TDP-43 oligomers can reproduce this observed cell type-specific toxicity, providing further support to a protein oligomer-mediated toxicity hypothesis in ALS.

## Materials and methods

Detailed methods are provided in the Supplementary material.

### Preparation of post-mortem tissue extracts

Initial sarkosyl-insoluble extracts from frozen CNS tissue for seeding were prepared as previously described (Smethurst *et al.*, 2016). See Supplementary material for more detailed information.

### Induced pluripotent stem cell culture, motor neuron and astrocyte differentiation

IPSCs were maintained using standard protocols. Motor neuron and astrocyte differentiation were carried out as described previously (Hall *et al.*, 2017; Simone *et al.*, 2017; Tyzack *et al.*, 2017; Luisier *et al.*, 2018). See Supplementary material for more detailed information.

### Data availability

Data supporting the findings of this study are available from the corresponding authors, upon reasonable request.

## Results

### Seeded aggregation of TDP-43 reveals a motor neuron-specific vulnerability

We have previously demonstrated seeded aggregation in TDP-43 overexpressing HEK293 cells by treating with spALS extract from post-mortem tissue from multiple cases that was not observed with age-matched controls (Smethurst *et al.*, 2016). We first attempted to determine a proof-of-principle by seeding from spALS spinal cord extract in HEK293 cells in the absence of TDP-43 overexpression. We previously established that 5 µg of material from the ALS insoluble extracts was sufficient to induce seeded aggregation (Smethurst *et al.*, 2016). Furthermore, it has also been shown that dose dependent increases in the seeded aggregation reaction plateau at 5 µg, with 10 µg of material not producing any significant increase in TDP-43 pathology (Nonaka *et al.*, 2013). Therefore, for all experiments we used 5 µg per treatment. We transfected spALS extract using Lipofectamine 3000™ or PULSin reagents. Post-transfection we detected formation of cytoplasmic inclusions of TDP-43 with simultaneous nuclear clearing of endogenous TDP-43 with spALS extracts. We confirmed that this seeded aggregation reaction was not attributable to the extraction procedure itself by transfecting control spinal cord extract, which demonstrated no detectable TDP-43 pathology (Supplementary Fig. 1).

**Figure 1:**
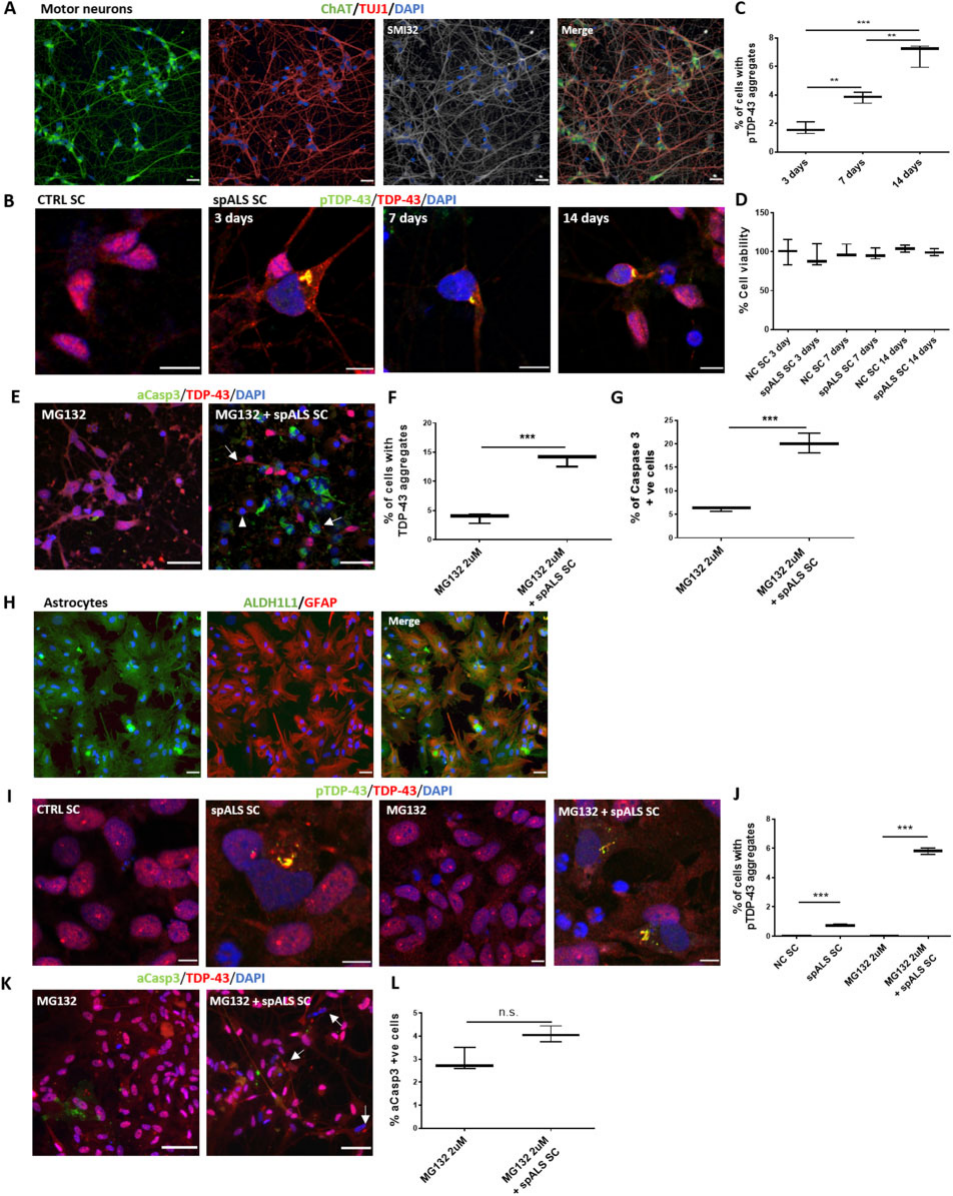
**TDP-43 seeded aggregation in human iPSC-derived motor neurons and astrocytes.** (**A**) Representative images of human iPSC motor neurons immunolabelled with ChAT (green), beta III tubulin (TUJ1, red), and SMI32 (white). Scale bars = 100 µm. (**B**) Representative images of seeded aggregation paradigm in human iPSC-derived motor neurons using normal control spinal cord (CTRL SC) or spALS spinal cord extract at 3, 7 and 14 days immunolabelled for pTDP-43 (green), TDP-43 (red), and counterstained with DAPI (blue). Scale bars = 10 μm. (**C**) Quantification of percentage of cells containing pTDP-43-positive aggregates in human iPSC-derived motor neurons at 3, 7 and 14 days. (**D**) MTT assay of cellular viability in motor neurons transfected with either CTRL spinal cord or spALS spinal cord for 3-, 7- and 14-day time points. (**E**) Representative images of motor neuron cultures treated with proteasome inhibitor MG132 alone (2 µM, 6 h) or MG132 + spALS spinal cord stained for nuclei (DAPI, blue), TDP-43 (red) and activated Casp3 (green). Arrows indicate TDP-43 aggregates and arrowheads represent motor neurons with nuclear cleared TDP-43. Scale bars = 30 μm. (**F**) Quantification of percentage of cells containing TDP-43 aggregates upon treatment with MG132 alone and MG132 plus spALS spinal cord extract. (**G**) Quantification of activated Casp3-positive cells per total cell number at each treatment condition. (**H**) Representative images of human iPSC-derived astrocytes immunolabelled for ALDH1L1 (green) and GFAP (red). Scale bars = 100 µm. (**I**) Representative images of astrocytes with either no treatment, treated with CTRL SC, MG132 (2 µM, 6 h) alone and with MG132 plus transfection spALS spinal cord extract and fixed at 3 days stained with DAPI (blue), and immunolabelled for TDP-43 (red) and pTDP-43 (green). Scale bars = 30 µm. (**J**) Percentage of cells with pTDP-43 aggregates following treatment with either control spinal cord, spALS spinal cord, MG132 alone (2 µM, 6 h) and MG132 plus transfection of spALS spinal cord extract fixed at 3 days. (**K**) Representative images of astrocytes treated with MG132 (2 µM, 6 h) alone or with MG132 plus transfection of spALS spinal cord extract and fixed at 3 days stained with DAPI (blue), and immunolabelled for TDP-43 (red) and activated Casp3 (green). Scale bars = 30 µm. Arrows indicate TDP-43 aggregates and/or with nuclear clearance of TDP-43. (**L**) Percentage of cells with activated Casp3 following treatment of cultures with MG132 alone (2 µM, 6 h) or MG132 plus transfection of spALS spinal cord extract. Data are expressed as mean ± SEM and represent three separate control cell lines, each in technical triplicate **P < 0.01 and ***P < 0.001; n.s. = not significant.

We have previously reported highly efficient, comprehensively characterized and functionally validated methods of generating human iPSC-derived motor neurons (Hall *et al.*, 2017; Luisier *et al.*, 2018; Maffioletti *et al.*, 2018) that are positive for choline acetyl transferase (ChAT), SMI-32 and beta III tubulin (TUJ1) (Fig. 1A). We first confirmed that the extract preparation procedure was not sufficient in itself to induce seeded aggregation of TDP-43 by transfecting control spinal cord extract into motor neurons (Fig. 1B). We next attempted the same experiment using spALS spinal cord samples, which led to TDP-43 aggregation in motor neurons after 3 days as demonstrated by TDP-43 and phosphorylated TDP-43 (pTDP-43) co-positive inclusions, with nuclear clearing of endogenous TDP-43 (Fig. 1B). This process continued at 7- and 14-days post inoculation including round globular inclusions (Fig. 1B, Day 3 panel) and skein-like inclusions (Fig. 1B, Day 14 panel) that are morphologically representative of TDP-43 inclusions observed in ALS post-mortem tissue (Fig. 1B). The percentage of cells with TDP-43 inclusions increased from Day 3 (∼2%) to Day 7 (∼3.8%, *P < *0.01) and from Days 7 to 14 (6.7%, *P < *0.01) (Fig. 1C). However, MTT assays revealed no decrease in cell viability (Fig. 1D).

To increase the abundance of seeded aggregation in motor neurons we used proteasomal inhibition to reduce clearance of TDP-43 aggregates and temporarily induce cytoplasmic TDP-43 mislocalization (Urushitani *et al.*, 2010; Wang *et al.*, 2010; van Eersel *et al.*, 2011; Scotter *et al.*, 2014). However, upon initial treatment with MG132 alone, we detected a large decrease in cell viability when treating with 5 µM for 3 days (data not shown) and therefore further optimized the concentration to 2 µM for 6 h. By subsequently adding our seeded aggregation paradigm to these cultures, we demonstrated that the number of aggregates significantly increased after 3 days (∼15%; *P < *0.001) compared to treatment with the proteasome inhibition alone (∼4%) (Fig. 1E and F). We also observed a significant increase in activated caspase 3 (Casp3) immunolabelling (∼20%, *P < *0.01) compared to proteasome inhibition alone (∼6%) (Fig. 1G), suggesting that the seeded aggregation process is toxic to motor neurons in the context of impaired proteosomal function. We next sought to address if astrocytes differed in their response to this seeded aggregation paradigm. Human iPSC-derived astrocytes were generated using our established and highly enriched platform (Hall *et al.*, 2017; Tyzack *et al.*, 2017; Thelin *et al.*, 2020). Noting that astroglial markers such as glial fibrillary acidic protein (GFAP) vary in expression depending on regional identity, activation state and ageing, we used the reliable general CNS astrocyte marker aldehyde dehydrogenase 1 family member L1 (ALDH1L1) in addition to GFAP (Fig. 1H). Treatment with spALS spinal cord extract demonstrated a small but significant number of pTDP-43 and TDP-43 co-positive inclusions (∼0.75%, *P < *0.001) (Fig. 1I and J) with endogenous nuclear TDP-43 clearance. We again confirmed that seeded aggregation of TDP-43 was not attributable to the extraction procedure itself by transfecting control spinal cord extract into astrocytes (Fig. 1I). After pre-treatment of human iPSC-derived astrocytes with MG132 (6 h at 2 µM) and subsequent transfection of spALS spinal cord extract, numerous TDP-43-positive inclusions formed after 3 days (Fig. 1J). The frequency of these TDP-43 aggregates was significantly higher (∼5.8%, *P < *0.01) than treatment with MG132 alone (Fig. 1J). However, unlike in motor neurons, no significant increase in apoptosis was detected indicating that astrocytes may be comparatively resilient to our seeded aggregation paradigm (Fig. 1K and L).

### TDP-43 aggregates preferentially spread from motor neurons to astrocytes

**Figure 2:**
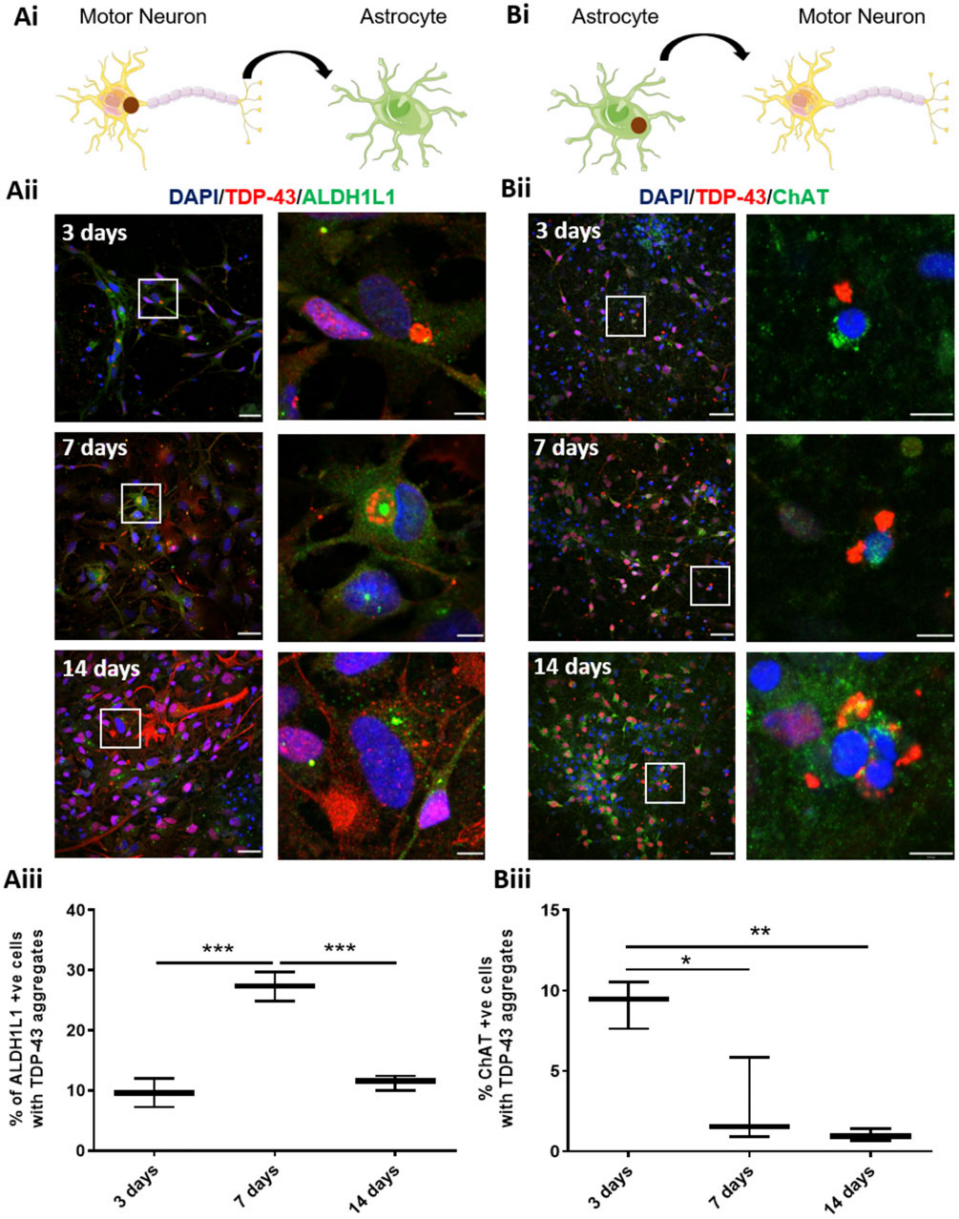
**Spread and propagation of TDP-43 aggregates between motor neurons and astrocytes.** [**A**(**i**–**iii**)] Motor neuron to astrocyte co-culture. [**A**(**i**)] Diagram of a motor neuron with TDP-43 aggregate transfer to an astrocyte. [**A**(**ii**)] Representative images of motor neurons seeded with TDP-43 aggregates and co-cultured for 3 (*top*), 7 (*middle*), and 14 days (*bottom*) with non-treated healthy astrocytes. White *inset* box is displayed to the *right* at high power magnification. Blue = DAPI; red = TDP-43; green = ALDH1L1. Scale bars = 50 µm in the *left column*; 10 µm in the *right column*. [**A**(**iii**)] Percentage of ALDH1L1-positive astrocytes containing TDP-43 aggregates at 3, 7 and 14 days. [**B**(**i**–**iii**)] Motor neuron to astrocyte co-culture. [**B**(**i**)] Diagram of an astrocyte with TDP-43 aggregate transfer to a motor neuron. [**B**(**ii**)] Representative images of astrocytes seeded with TDP-43 aggregates and co-cultured for 3 (*top*), 7 (*middle*), and 14 days (*bottom*) with healthy non-treated motor neurons. White *inset* box is displayed to the *right* at high power magnification. Blue = DAPI; red = TDP-43; green = ChAT. Scale bars = 50 µm in the *left column*; 10 µm in the *right column*. [**B**(**iiii**)] Percentage of ChAT-positive motor neurons containing TDP-43 aggregates at 3, 7 and 14 days. Data are expressed as mean ± SEM and represent three separate control cell lines, each in technical triplicate, *P < 0.05 **P < 0.01 and ***P < 0.001. Panels **A**(**i**) and **B**(**i**) were constructed using images from free-to-use resources at https://smart.servier.com/.

To determine if TDP-43 aggregates could spread between motor neurons and astrocytes, we cultured control untreated astrocytes together with motor neurons that had been treated with our optimized seeded aggregation paradigm described above for 3, 7 and 14 days [Fig. 2A(i)]. We first confirmed that conditioned medium from cultures exhibiting seeded aggregation could not induce this process in untreated cultures (data not shown), which is also consistent with our previous work (Smethurst *et al.*, 2016). Using this co-culture paradigm, we detected the formation of TDP-43-positive aggregates that also cleared endogenous nuclear TDP-43 in ALDH1L1-positive astrocytes [Fig. 2A(ii)]. The percentage of astrocytes containing TDP-43-positive aggregates significantly increased from 9.6% at Day 3 to 27.3% at Day 7 (*P < *0.001). After this time point the percentage of astrocytes containing TDP-43-positive aggregates significantly decreased from 27.3% to 11.3% (*P < *0.001) indicating a clearance or dilution of TDP-43 aggregates over time [Fig. 2A(iii)].

Next, we investigated the propagation of TDP-43 aggregates from astrocytes to motor neurons by performing seeded aggregation in astrocytes and then co-culturing with control untreated motor neurons for 3, 7 and 14 days [Fig. 2B(i)]. We observed ChAT-positive motor neurons containing TDP-43 aggregates with cleared nuclear endogenous TDP-43 at 3 days [Fig. 2B(ii)], indicating that TDP-43 aggregates can propagate from astrocytes to motor neurons. At Day 7, the number of aggregates in ChAT-positive cells decreased significantly from 9.2% to 2.8% (*P < *0.05) and again to 1% at Day 14 (*P < *0.01) [Fig. 2B(iii)]. These data indicate that TDP-43 aggregates can initially propagate from astrocytes to motor neurons but they are cleared after 7 and 14 days. Cumulatively, these data from iterative co-culture paradigms raise the possibility that astrocytes may, at least initially, exhibit neuroprotective properties in the context of seeded TDP-43 proteinopathy.

### Astrocytes protect motor neurons from seeded TDP-43 aggregation and toxicity

**Figure 3:**
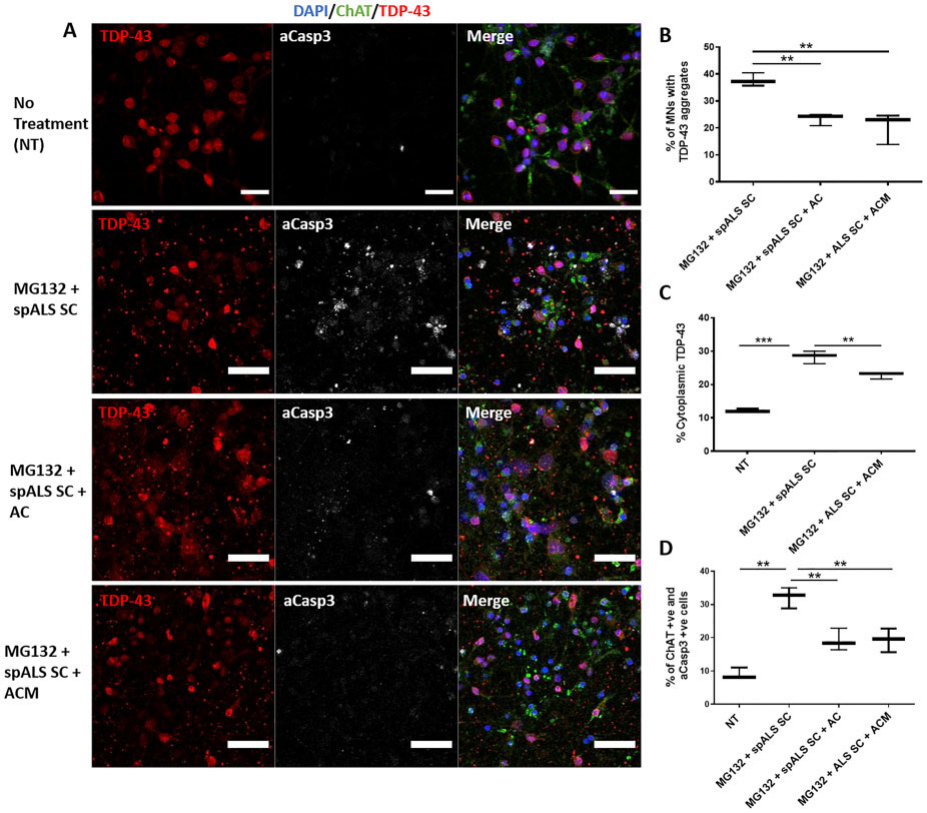
**Astrocytes are neuroprotective to seeded aggregation and toxicity in motor neurons.** (**A**) Representative images of motor neurons with no treatment, MG132 + spALS spinal cord, MG132 + spALS spinal cord + astrocyte, MG132 + spALS spinal cord + ACM triple immunolabelled for the motor neuron marker ChAT (green), TDP-43 (red) activated Casp3 (white) and counterstained with DAPI (blue). Scale bars = 30 µm. (**B**) Quantification of percentage of ChAT-positive cells containing TDP-43 aggregates in each treatment. (**C**) Percentage of cytoplasmic TDP-43 in motor neurons in each treatment. (**D**) Percentage of ChAT-positive and activated Casp3-positive cells in each treatment. Data are expressed as mean ± SEM and represent three separate control cell lines, each in technical triplicate, *P < 0.05 **P < 0.01 and ***P < 0.001.

To investigate the neuroprotective capacity of human astrocytes, we took motor neurons after seeded aggregation for 3 days and performed a co-culture experiment. We quantified the number of ChAT-positive motor neurons with TDP-43 aggregates and found that both co-culture and astrocyte conditioned medium (ACM) treatments significantly reduced the percentage of cells containing TDP-43-positive aggregates from 37.8% to 23.4% (astrocyte co-culture) (*P < *0.01) and 20.4% (ACM) (*P < *0.01) (Fig. 3A and B). Crucially, treatment with ACM also reduced cytoplasmic TDP-43 in motor neurons from 28.3% to 22.7% (*P < *0.01) (Fig. 3C). Finally, we a found a significant reduction in the number of activated Casp3-positive cells from 32.2% to 19.2% (*P < *0.01) with astrocyte co-culture and 32.2% to 19.3% (*P < *0.01) with ACM treatment (Fig. 3D). These data suggest astrocyte-mediated neuroprotection, which is—at least in part—contact independent. This neuroprotective astrocyte state in our paradigm is further reinforced by the absence of expression of complement 3 (C3), a recognized marker of toxic astrocytes (Liddelow *et al.*, 2017) (Supplementary Fig. 2A).

### Recombinant TDP-43 oligomers are toxic to motor neurons but not astrocytes

**Figure 4:**
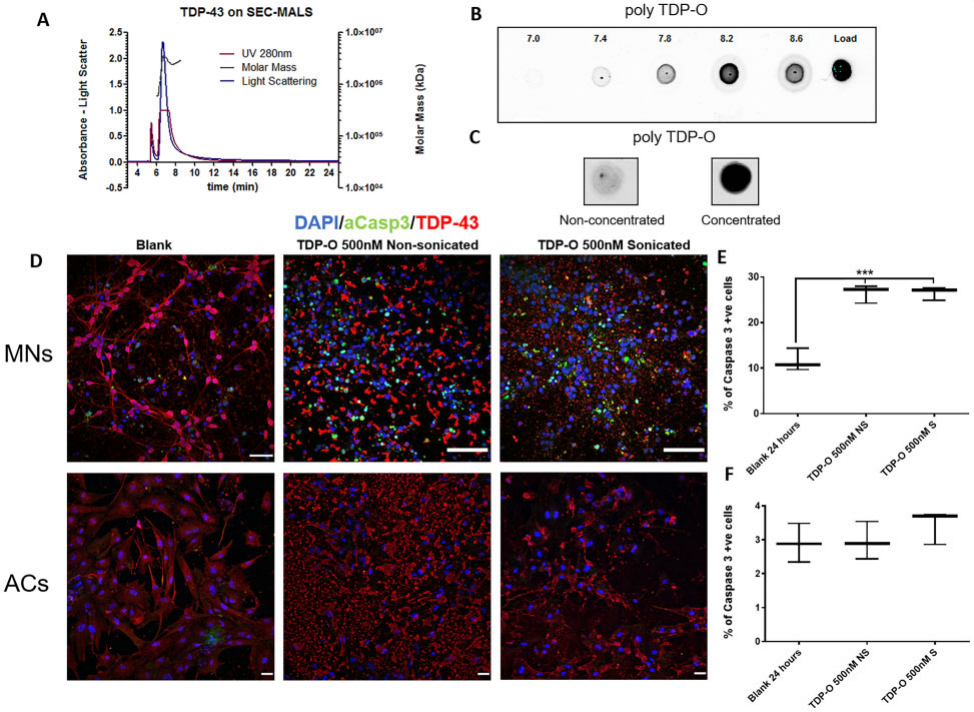
**TDP-43 oligomers are comparatively more toxic to motor neurons when compared to astrocytes.** (**A**) SEC-MALS analysis of recombinant TDP-43 purified from HEK cells demonstrating high molecular weight between elution fractions 6–8 min against absorbance at 280 nm (red line), Molar mass in kDa (green line), and light scattering (blue line). (**B**) Dot blot of fractions under the high molecular weight peak (7–8.6 min) immunolabelled with polyclonal TDP-43 oligomer antibody. Load fraction was whole fraction pre-SEC. (**C**) Dot blot of fraction 8.2 that was subject to concentration. (**D**) *Top row:* Representative images of motor neurons (MNs) treated with non-sonicated or sonicated 500 nM TDP oligomer for 24 h and stained with DAPI (blue), and immunolabelled for TDP-43 (red), and activated Casp3 (green). Scale bars = 50 µm. *Bottom row*: Representative images of astrocytes (ACs) treated with non-sonicated or sonicated 500 nM TDP-43 oligomer for 24 h and stained with DAPI (blue), and immunolabelled for TDP-43 (red), and activated Casp3 (green). Scale bars = 90 mm. (**E**) Quantification of activated Casp3-positive motor neurons after 24 h treated with sonicated or non-sonicated TDP-43 oligomer for 24 h. (**F**) Quantification of activated Casp3-positive astrocytes after 24-h treatment with 500 nM of either non-sonicated or sonicated TDP-43 oligomer for 24 h. Data are expressed as mean ± SEM and represent three separate control cell lines, each in technical triplicate.

There is now accumulating evidence to support the notion that soluble oligomeric species of misfolded proteins are the cause of toxicity in several neurodegenerative diseases with prominent protein misfolding (Benilova *et al.*, 2012; Forloni *et al.*, 2016). Having previously demonstrated that our paradigm using sarkosyl-insoluble extract from post-mortem ALS tissue can induce the formation of TDP-43 oligomers in HEK293 cells (Smethurst *et al.*, 2016), we first confirmed the presence of TDP-43 oligomer in spALS spinal cord extract itself using a previously characterized TDP-43 oligomer-specific antibody to probe dot blots (Supplementary Fig. 3). We next tested the hypothesis that it was TDP-43 oligomers themselves that were (at least part of) the toxic principle. To address this we prepared recombinant TDP-43 oligomer expressed in HEK293 cells. This purified recombinant TDP-43 was then subject to analytical size exclusion chromatography, coupled with multi-angle light scattering (SEC-MALS) where the majority of the TDP-43 was detected in the void volume (high molecular weight fraction) with an average molecular mass of 2000 kDa indicating that TDP-43 readily forms large aggregates in a purified state (Fig. 4A). To check for the presence of TDP-43 oligomers in these fractions we used TDP-43 oligomer-specific antibody to probe dot blots of fractions taken under the high molecular weight peak (fractions 7–8.6). We detected the strongest signal for TDP-43 oligomer in the 8.2 fraction (Fig. 4B). To obtain large amounts of concentrated TDP-43 oligomer for experimental use we increased the amount of recombinant protein generated from the HEK293s and performed SEC. This protein was eluted in a high volume of buffer and then subject to a centrifugal filter to produce a more concentrated preparation of TDP-43 oligomers (∼70 mM) (Fig. 4C).

To investigate cell type-specific toxicity of TDP-43 oligomers we applied them to cultures of human iPSC-derived motor neurons and astrocytes, using previously determined toxic concentrations (500 nM) for 24 h (Fang *et al.*, 2014). Additionally, we sonicated these preparations to break down any potentially larger species to determine if this conferred any difference in toxicity. We demonstrated that these oligomers were significantly toxic to motor neurons in both sonicated and non-sonicated forms (26.5%, *P < *0.001) compared to the control (11.6%; lower molecular weight fraction of the SEC with TDP-43 oligomer absent) after 24 h, detected via an increase in activated Casp3 immunolabelling (Fig. 4D, top and E). Interestingly, these oligomers were comparatively less toxic to astrocytes under the same treatment conditions (Fig. 4D and F) and did not induce C3 expression (Supplementary Fig. 2B). These data further support the cell type-specific differences observed in our seeded aggregation paradigm and suggest that toxicity in this context is likely, at least in part, attributable to TDP-43 oligomer species.

## Discussion

We have demonstrated that seeded aggregation can successfully be achieved in HEK293 cultures without overexpressing TDP-43, using enriched seeding material. We next applied this to human iPSC-derived motor neurons. Indeed, most studies of *in vitro* prion-like seeded aggregation of proteins such as tau (Frost *et al.*, 2009*a*, *b*; Nonoka *et al.*, 2010; Guo and Lee, 2011), SOD1 (Münch *et al.*, 2011; Furukawa *et al.*, 2013) and TDP-43 (Furukawa *et al.*, 2011; Nonaka *et al.*, 2013) used an overexpression background to facilitate seeding, which our model circumvents, therefore representing a more physiological human model of TDP-43 pathology for studying sporadic ALS in the target cell type.

The number of TDP-43 aggregates reproducibly and significantly increased in frequency with longer incubations, which is consistent with either *de novo* formation over these time periods and/or aggregate spreading from cell-to-cell. We have not formally excluded the possibility that these findings result from a time-dependent increase in internalized persistent aggregates from the ALS inocula, although all cultures were routinely washed rigorously with fresh medium three times 6 h after transfection to mitigate this risk. Future studies might systematically address this through molecular labelling of the seeds coupled with live cell imaging. A significant increase in the seeded aggregation reaction was observed upon treatment with MG132 (∼15% versus ∼2% at Day 3). We have previously demonstrated that serial passage of TDP-43 pathology further enhances its potency and the increased abundance of seeded aggregation demonstrated in the experiments performed in Fig. 3 compared to those in Fig. 1 reproduces this earlier published finding (Smethurst *et al.*, 2016).

The lack of toxicity in human iPSC-derived motor neurons treated with seeded aggregation alone in this study likely reflects low frequency and abundance of TDP-43 inclusions observed. However, upon proteasomal inhibition and subsequent seeded aggregation, the number of TDP-43 aggregates and activated Casp3-positive cells significantly increased compared to proteasome inhibition alone suggesting that such TDP-43 seeded aggregation is indeed toxic under defined cellular states. The sequential proteasome inhibition and inoculation of the spALS spinal cord extract resulted in the increased formation of TDP-43 aggregates in both human iPSC motor neurons and astrocytes. Indeed, a mouse motor neuron-driven expression of proteasome dysfunction, but not autophagy, resulted in a robust model of TDP-43, FUS, ubiquilin-2 and optineurin mislocalization and aggregation with a resultant ALS phenotype (Tashiro *et al.*, 2012).

The number of TDP-43 aggregates is higher in all treatment conditions in motor neurons compared to astrocytes suggesting that motor neurons are more susceptible to proteasome-induced exacerbated seeding of TDP-43 than astrocytes. This is also supported by evidence from the differential over expression of TDP-43 in motor neurons and astrocytes in mouse brain, where neurons were more vulnerable to TDP-43 pathology initially but ageing increased the accumulation of TDP-43 in astrocytes, and inhibition of the proteasome caused an increase in astrocyte-mediated toxicity (Sen *et al.*, 2013). Spreading and propagation of pTDP-43 aggregates *in vitro* via exosomes and along neuronal processes have been previously established (Nonaka *et al.*, 2013; Feiler *et al.*, 2015). TDP-43 proteinopathy spread form motor neuron to astrocyte and astrocyte to motor neuron most likely also uses these same mechanisms. Indeed, evidence from prion studies shows that propagation of the scrapie isoform of prion protein (PrP^Sc^) in primary neuron and astrocytes co-culture happens in a cell contact dependent manner via tunnelling nanotubes (Victoria *et al.*, 2016). Astrocytes are proposed to have a higher efficiency of protein clearance than neurons (Tydlacka *et al.*, 2008) and this may be an intrinsic protection mechanism of astrocytes to clear pathology from neurons. We speculate that a ‘help me’ signal from motor neurons to astrocytes could ultimately (once juxtaposed astrocytes signal back) lead to the release of toxic TDP-43 aggregates from motor neurons for their subsequent uptake and clearance within astrocytes, which is consistent with our data. More recent evidence supporting our data shows that α-synuclein fibrils and aggregates are efficiently spread between primary neurons and astrocytes and between astrocytes themselves, but less so from astrocytes to neurons. Astrocytes were found to be more efficient at degrading α-synuclein aggregates than neurons (Loria *et al.*, 2017). Similar phenomena have also been noted for prions in primary cultures (Choi *et al.*, 2014) and for huntingtin aggregates in *Drosophila* (Pearce *et al.*, 2015). Therefore, the further elucidation of these mechanisms could lead to protective therapeutic avenues that limit spread and increase protein degradation via manipulation of neuroprotective astrocyte responses. These differences in propagation and spread may well also be dependent on the strain of the pathological misfolded protein and/or differential endocytic activity between the two cell types. These potential mechanisms warrant further investigation to understand the complex cell type-specific biology of TDP-43 spread and propagation. Additionally, it is noteworthy that (unlike motor neurons) astrocytes can still proliferate upon reactive transformation (Barres and Barde, 2000), which could allow them to dilute out any cytotoxic misfolded proteins by means of cell division. The relative resilience of astrocytes to seeded aggregation might also be explained by ubiquitin–proteasome system activity being higher in glia than neurons, resulting in their selective vulnerability to perturbed proteostasis (Tydlacka *et al.*, 2008).

Previous studies have indicated non-cell autonomous deleterious effects by astrocytes carrying mutations in *TARDBP* (Tong *et al.*, 2013), *SOD1* (Nagai *et al.*, 2007; Di Giorgio *et al.*, 2008; Marchetto *et al.*, 2008; Rojas *et al.*, 2014), *C9orf72* (Madill *et al.*, 2017) and *VCP* (Hall *et al.*, 2017). Furthermore astrocytes from patients with sporadic ALS were also found to be deleterious (Re *et al.*, 2014). In contrast to studying astrocytes derived from patients with ALS, we have studied the responses of wild-type astrocytes to sporadic ALS-related pathomechanisms. The lack of deleterious astrocyte reactive transformation we observed here further underpins the resilient nature of astrocytes to form a neuroprotective response to early insults. Therefore, we would speculate that our studies represent very initial (preclinical) responses of astrocytes to sporadic ALS at disease onset. We propose that astrocytes may undergo deleterious A1 reactive transformation later in the disease process (Liddelow *et al.*, 2017), but may well adopt compensatory A2 responses (Anderson *et al.*, 2016) at the earliest preclinical stage. However, these hypotheses clearly warrant further investigation from follow-up studies.

Here we have demonstrated the efficient production, purification and concentration of TDP-43 oligomers that are subsequently toxic to human motor neurons but not astrocytes. These data provide further evidence for the toxicity of the oligomeric species of aggregated misfolded proteins in ALS (Benilova *et al.*, 2012). They also confirm the previous findings of toxicity of these species in primary cortical neurons and *in vivo* injections of wild type mice (Fang *et al.*, 2014) and the presence of these oligomers in FTLD and ALS tissue (Kao *et al.*, 2015). Here, however, we were able to demonstrate significant specific toxicity of these oligomers in human motor neurons further confirming neuronal susceptibility. A prominent hypothesis for protein oligomer toxicity is the interaction with lipids in membranes including the formation of membrane permeable pores (Andreasen *et al.*, 2015) and ion channels (Bode *et al.*, 2017). Other potential mechanisms include proteasome impairment, mitochondrial dysfunction, alteration of signalling pathways, disruption of synaptic signalling and inhibition of autophagy (Kayed and Lasagna-Reeves, 2012). However, the exact mechanisms of TDP-43 oligomer toxicity are currently unknown. The resilience of astrocytes to both TDP-43 oligomer treatment and seeded aggregation observed here is intriguing and may be due to lack of cellular uptake of the oligomers, more efficient protein clearance machinery in astrocytes and potential neuronal receptor dependent mechanisms of toxicity. Our co-culture experiments clearly demonstrate that astrocytes are, at least initially, neuroprotective to seeded aggregation within motor neurons by reducing TDP-43 proteinopathy and toxicity. Therefore, our work raises the prospect of invoking/harnessing endogenous neuroprotective features of astrocytes as a therapeutic target in ALS.

## Funding

This work was supported by the Francis Crick Institute which receives its core funding from Cancer Research UK (FC010110), the UK Medical Research Council (FC010110), and the Wellcome Trust (FC010110). R.P. holds an MRC/MND Association Lady Edith Wolfson Senior Clinical Fellowship (MR/S006591/1) and is supported by the National Institute for Health Research University College London Hospitals Biomedical Research Centre.

## Competing interests

The authors report no competing financial interests.

## Supplementary Material

awz419_Supplementary_Information

## Abbreviations

ALS =: amyotrophic lateral sclerosis
ChAT =: choline acetyl transferase
iPSC =: induced pluripotent stem cell
spALS =: serially passaged sporadic ALS post-mortem tissue

## References

[awz419-B1] Al-Chalabi A, Hardiman O The epidemiology of ALS: a conspiracy of genes, environment and time. Nat Rev Neurol 2013; 9: 617–62. 24126629 10.1038/nrneurol.2013.203

[awz419-B2] Anderson MA, Burda JE, Ren Y, Ao Y, O’Shea TM, Kawaguchi R, et al Astrocyte scar formation aids central nervous system axon regeneration. Nature 2016; 532: 195–200. 27027288 10.1038/nature17623 PMC5243141

[awz419-B3] Andreasen M, Lorenzen N, Otzen D Interactions between misfolded protein oligomers and membranes: a central topic in neurodegenerative diseases? Biochim Biophys Acta 2015; 1848: 1897–907. 25666871 10.1016/j.bbamem.2015.01.018

[awz419-B4] Arai T, Hasegawa M, Akiyama H, Ikeda K, Nonaka T, Mori H, et al TDP-43 is a component of ubiquitin-positive tau-negative inclusions in frontotemporal lobar degeneration and amyotrophic lateral sclerosis. Biochem Biophys Res Commun 2006; 351: 602–11. 17084815 10.1016/j.bbrc.2006.10.093

[awz419-B5] Barres BA, Barde Y-A Neuronal and glial cell biology. Curr Opin Neurobiol 2000; 10: 642–8. 11084327 10.1016/s0959-4388(00)00134-3

[awz419-B6] Benilova I, Karran E, De Strooper B The toxic Aβ oligomer and Alzheimer’s disease: an emperor in need of clothes. Nat Neurosci 2012; 15: 349–57. 22286176 10.1038/nn.3028

[awz419-B7] Bode DC, Baker MD, Viles JH Ion channel formation by amyloid-β _42_ oligomers but not amyloid-β _40_ in cellular membranes. J Biol Chem 2017; 292: 1404–13. 27927987 10.1074/jbc.M116.762526 PMC5270483

[awz419-B101] Choi YP, Head MW, Ironside JW, Priola SA Uptake and degradation of protease-sensitive and -resistant forms of abnormal human prion protein aggregates by human astrocytes. Am J Pathol 2014; 184: 3299–307. 25280631 10.1016/j.ajpath.2014.08.005 PMC4258502

[awz419-B9] Collinge J Mammalian prions and their wider relevance in neurodegenerative diseases. Nature 2016; 539: 217–26. 27830781 10.1038/nature20415

[awz419-B10] Collinge J, Clarke AR A general model of prion strains and their pathogenicity. Science 2007; 318: 930–6. 17991853 10.1126/science.1138718

[awz419-B102] Di Giorgio FP, Boulting GL, Bobrowicz S, Eggan KC Human embryonic stem cell-derived motor neurons are sensitive to the toxic effect of glial cells carrying an ALS-causing mutation. Cell Stem Cell 2008; 3: 637–48. 19041780 10.1016/j.stem.2008.09.017

[awz419-B11] Fang Y-S, Tsai K-J, Chang Y-J, Kao P, Woods R, Kuo P-H, et al Full-length TDP-43 forms toxic amyloid oligomers that are present in frontotemporal lobar dementia-TDP patients. Nat Commun 2014; 5: 4824. 25215604 10.1038/ncomms5824

[awz419-B12] Feiler MS, Strobel B, Freischmidt A, Helferich AM, Kappel J, Brewer BM, et al TDP-43 is intercellularly transmitted across axon terminals. J Cell Biol 2015; 211: 897–911. 26598621 10.1083/jcb.201504057 PMC4657165

[awz419-B13] Forloni G, Artuso V, La Vitola P, Balducci C Oligomeropathies and pathogenesis of Alzheimer and Parkinson’s diseases. Mov Disord 2016; 31: 771–81. 27030592 10.1002/mds.26624

[awz419-B14] Frost B, Jacks RL, Diamond MI Propagation of tau misfolding from the outside to the inside of a cell. J Biol Chem2009a; 284: 12845–52. 19282288 10.1074/jbc.M808759200 PMC2676015

[awz419-B15] Frost B, Ollesch J, Wille H, Diamond MI Conformational diversity of wild-type tau fibrils specified by templated conformation change. J Biol Chem2009b; 284: 3546–51. 19010781 10.1074/jbc.M805627200 PMC2635036

[awz419-B16] Furukawa Y, Kaneko K, Watanabe S, Yamanaka K, Nukina N A seeding reaction recapitulates intracellular formation of sarkosyl-insoluble TAR DNA binding protein-43 inclusions. J Biol Chem 2011; 286: 18664–72. 21454603 10.1074/jbc.M111.231209 PMC3099683

[awz419-B17] Furukawa Y, Kaneko K, Watanabe S, Yamanaka K, Nukina N Intracellular seeded aggregation of mutant Cu, Zn-superoxide dismutase associated with amyotrophic lateral sclerosis. FEBS Lett 2013; 587: 2500–5. 23831581 10.1016/j.febslet.2013.06.046

[awz419-B18] Guo JL, Lee V Seeding of normal tau by pathological tau conformers drives pathogenesis of Alzheimer-like tangles. J Biol Chem 2011; 286: 1–27. 21372138 10.1074/jbc.M110.209296 PMC3083182

[awz419-B19] Hall CE, Yao Z, Choi M, Tyzack GE, Serio A, Luisier R, et al Progressive motor neuron pathology and the role of astrocytes in a human stem cell model of VCP-related ALS. Cell Rep 2017; 19: 1739–49. 28564594 10.1016/j.celrep.2017.05.024 PMC5464993

[awz419-B20] Kao PF, Chen Y-R, Liu X-B, DeCarli C, Seeley WW, Jin L-W Detection of TDP-43 oligomers in frontotemporal lobar degeneration-TDP. Ann Neurol 2015; 78: 211–21. 25921485 10.1002/ana.24431 PMC4822421

[awz419-B21] Kayed R, Lasagna-Reeves CA Molecular mechanisms of amyloid oligomers toxicity. J Alzheimers Dis 2012; 33: S67–S78. 10.3233/JAD-2012-129001 22531422

[awz419-B22] Liddelow SA, Guttenplan KA, Clarke LE, Bennett FC, Bohlen CJ, Schirmer L, et al Neurotoxic reactive astrocytes are induced by activated microglia. Nature 2017; 541: 481–7. 28099414 10.1038/nature21029 PMC5404890

[awz419-B103] Loria F, Vargas JY, Bousset L, Syan S, Salles A, Melki R, et al α-Synuclein transfer between neurons and astrocytes indicates that astrocytes play a role in degradation rather than in spreading. Acta Neuropathol 2017; 134: 789–808. 28725967 10.1007/s00401-017-1746-2

[awz419-B23] Luisier R, Tyzack GE, Hall CE, Mitchell JS, Devine H, Taha DM, et al Intron retention and nuclear loss of SFPQ are molecular hallmarks of ALS. Nat Commun 2018; 9: 2010. 29789581 10.1038/s41467-018-04373-8 PMC5964114

[awz419-B104] Madill M, McDonagh K, Ma J, Vajda A, McLoughlin P, O'Brien T, et al Amyotrophic lateral sclerosis patient iPSC-derived astrocytes impair autophagy via non-cell autonomous mechanisms. Mol Brain 2017; 10: 22. 28610619 10.1186/s13041-017-0300-4 PMC5470320

[awz419-B105] Maffioletti SM, Sarcar S, Henderson ABH, Mannhardt I, Pinton L, Moyle LA, et al Three-dimensional human iPSC-derived artificial skeletal muscles model muscular dystrophies and enable multilineage tissue engineering. Cell Rep 2018; 23: 899–908. 29669293 10.1016/j.celrep.2018.03.091 PMC5917451

[awz419-B106] Marchetto MCN, Muotri AR, Mu Y, Smith AM, Cezar GG,, Gage FH Non-cell-autonomous effect of human SOD1G37R astrocytes on motor neurons derived from human embryonic stem cells. Cell Stem Cell 2008; 3: 649–57. 19041781 10.1016/j.stem.2008.10.001

[awz419-B24] Münch C, O’Brien J, Bertolotti A Prion-like propagation of mutant superoxide dismutase-1 misfolding in neuronal cells. Proc Natl Acad Sci USA 2011; 108: 3548–53. 21321227 10.1073/pnas.1017275108 PMC3048161

[awz419-B107] Nagai M, Re DB, Nagata T, Chalazonitis A, Jessell TM, Wichterle H, et al Astrocytes expressing ALS-linked mutated SOD1 release factors selectively toxic to motor neurons. Nat Neurosci 2007; 10: 615–22. 17435755 10.1038/nn1876 PMC3799799

[awz419-B25] Neumann M, Sampathu DM, Kwong LK, Truax AC, Micsenyi MC, Chou TT, et al Ubiquitinated TDP-43 in frontotemporal lobar degeneration and amyotrophic lateral sclerosis. Science 2006; 314: 130–3. 17023659 10.1126/science.1134108

[awz419-B26] Nonaka T, Masuda-Suzukake M, Arai T, Hasegawa Y, Akatsu H, Obi T, et al Prion-like properties of pathological TDP-43 aggregates from diseased brains. Cell Rep 2013; 4: 124–34. 23831027 10.1016/j.celrep.2013.06.007

[awz419-B27] Nonoka T, Watanabe ST, Iwatsubo T, Hasegawa M Seeded aggregation and toxicity of alpha-synuclein and tau. J Biol Chem 2010; 285: 34885–98. 20805224 10.1074/jbc.M110.148460 PMC2966103

[awz419-B108] Pearce MMP, Spartz EJ, Hong W, Luo L, Kopito RR Prion-like transmission of neuronal huntingtin aggregates to phagocytic glia in the Drosophila brain. Nat Commun 2015; 6: 6768. 25866135 10.1038/ncomms7768 PMC4515032

[awz419-B109] Re DB, Le Verche V, Yu C, Amoroso MW, Politi KA, Phani S, et al Necroptosis drives motor neuron death in models of both sporadic and familial ALS. Neuron 2014; 81: 1001–8. 24508385 10.1016/j.neuron.2014.01.011 PMC3951532

[awz419-B110] Rojas F, Cortes N, Abarzua S, Dyrda A, van Zundert B Astrocytes expressing mutant SOD1 and TDP43 trigger motoneuron death that is mediated via sodium channels and nitroxidative stress. Front Cell Neurosci 2014; 8: 24. 10.3389/fncel.2014.00024 PMC3916762 24570655

[awz419-B28] Scotter EL, Vance C, Nishimura AL, Lee Y-B, Chen H-J, Urwin H, et al Differential roles of the ubiquitin proteasome system (UPS) and autophagy in the clearance of soluble and aggregated TDP-43 species. J Cell Sci 2014; 127: 1263–78. 24424030 10.1242/jcs.140087 PMC3953816

[awz419-B29] Sen Y, Wang C-E, Wei W, Gaertig MA, Lai L, Li S, et al TDP-43 causes differential pathology in neuronal versus glial cells in the mouse brain. Hum Mol Genet 2013; 23: 2678–93. 24381309 10.1093/hmg/ddt662 PMC3990167

[awz419-B30] Simone R, Balendra R, Moens TG, Preza E, Wilson KM, Heslegrave A, et al G-quadruplex-binding small molecules ameliorate C9orf72 FTD/ALS pathology in vitro and in vivo. EMBO Mol Med 2017; 10: 22–31. 10.15252/emmm.201707850 PMC5760849 29113975

[awz419-B31] Smethurst P, Newcombe J, Troakes C, Simone R, Chen Y-R, Patani R, et al In vitro prion-like behaviour of TDP-43 in ALS. Neurobiol Dis 2016; 96: 236–47. 27590623 10.1016/j.nbd.2016.08.007 PMC5113659

[awz419-B32] Tashiro Y, Urushitani M, Inoue H, Koike M, Uchiyama Y, Komatsu M, et al Motor neuron-specific disruption of proteasomes, but not autophagy, replicates amyotrophic lateral sclerosis. J Biol Chem 2012; 287: 42984–94. 23095749 10.1074/jbc.M112.417600 PMC3522293

[awz419-B112] Thelin EP, Hall CE, Tyzack GE, Frostell A, Giorgi-Coll S, Alam A, et al Delineating astrocytic cytokine responses in a human stem cell model of neural trauma. J Neurotrauma 2020; 37: 93–105. 31452443 10.1089/neu.2019.6480 PMC6921298

[awz419-B111] Tong J, Huang C, Bi F, Wu Q, Huang B, Liu X, et al Expression of ALS-linked TDP-43 mutant in astrocytes causes non-cell-autonomous motor neuron death in rats. EMBO J 2013; 32: 1917–26. 23714777 10.1038/emboj.2013.122 PMC3981181

[awz419-B33] Tsuji H, Arai T, Kametani F, Nonaka T, Yamashita M, Suzukake M, et al Molecular analysis and biochemical classification of TDP-43 proteinopathy. Brain 2012; 135: 3380–91. 10.1093/brain/aws230 23035040

[awz419-B34] Tydlacka S, Wang C-E, Wang X, Li S, Li X-J Differential activities of the ubiquitin-proteasome system in neurons versus glia may account for the preferential accumulation of misfolded proteins in neurons. J Neurosci 2008; 28: 13285–95. 10.1523/JNEUROSCI.4393-08.2008 PMC2662777 19052220

[awz419-B35] Tyzack G, Lakatos A, Patani R Human Stem cell-derived astrocytes: specification and relevance for neurological disorders. Curr Stem Cell Rep 2016; 2: 236–47. 27547709 10.1007/s40778-016-0049-1 PMC4972864

[awz419-B36] Tyzack GE, Hall CE, Sibley CR, Cymes T, Forostyak S, Carlino G, et al A neuroprotective astrocyte state is induced by neuronal signal EphB1 but fails in ALS models. Nat Commun 2017; 8: 1164. 29079839 10.1038/s41467-017-01283-z PMC5660125

[awz419-B37] Urushitani M, Sato T, Bamba H, Hisa Y, Tooyama I Synergistic effect between proteasome and autophagosome in the clearance of polyubiquitinated TDP-43. J Neurosci Res 2010; 88: 784–97. 19798749 10.1002/jnr.22243

[awz419-B38] van Eersel J, Ke YD, Gladbach A, Bi M, Götz J, Kril JJ, et al Cytoplasmic accumulation and aggregation of TDP-43 upon proteasome inhibition in cultured neurons. PloS One 2011; 6: e22850. 21829535 10.1371/journal.pone.0022850 PMC3146516

[awz419-B113] Victoria GS, Arkhipenko A, Zhu S, Syan S, Zurzolo C Astrocyte-to-neuron intercellular prion transfer is mediated by cell-cell contact. Sci Rep 2016; 6: 20762. 26857744 10.1038/srep20762 PMC4746738

[awz419-B39] Walker LC, Jucker M Neurodegenerative diseases: expanding the prion concept. Annu Rev Neurosci 2015; 38: 87–103. 25840008 10.1146/annurev-neuro-071714-033828 PMC4803040

[awz419-B40] Wang X, Fan H, Ying Z, Li B, Wang H, Wang G Degradation of TDP-43 and its pathogenic form by autophagy and the ubiquitin-proteasome system. Neurosci Lett 2010; 469: 112–6. 19944744 10.1016/j.neulet.2009.11.055

[awz419-B41] Yamanaka K, Komine O The multi-dimensional roles of astrocytes in ALS. Neurosci Res 2018; 126: 31–8. 29054467 10.1016/j.neures.2017.09.011

