# Supplementary material for: Distinct responses of neurons and astrocytes to TDP-43 proteinopathy in amyotrophic lateral sclerosis
Source: Brain. 2020 Feb 10;143(2):430–40. doi: 10.1093/brain/awz419 (PMC7009461; doi:10.1093/brain/awz419)
Supplement: awz419_Supplementary_Information [file awz419_supplementary_information.pdf]

Supplementary information

Supplementary methods

Post-mortem tissue
Post-mortem spinal cord tissue samples were obtained from the NeuroResource tissue bank (UCL Queen Square Institute of Neurology, London). All tissue samples were obtained with informed consent and under NHS Research Ethics Committee and Human Tissue Authority (HTA) regulations. Importantly, we have previously demonstrated seeded aggregation from post-mortem tissue taken from multiple cases of ALS (but not from matched controls) (See Smethurst et al., 2016). These cases had clinically, electrophysiologically and pathologically definite ALS with no known family history or gene mutation (including C9orf72, TDP-43, SOD1 and FUS). Against this background, we utilised tissue extract from a representative case for the current in-depth study. This was a 71 year old female patient with a bulbar onset form of ALS. She eventually progressed to full limb involvement with cognitive decline and a diagnosis of frontotemporal dementia. This patient died 12 months after onset and post mortem neuropathological examination revealed widespread neuron loss and TDP-43 pathology in the motor cortex and spinal cord.

Preparation of post-mortem tissue extracts
Initial sarkosyl insoluble extracts from frozen CNS tissue for seeding were prepared as previously described (Smethurst et al., 2016). Briefly, 10% tissue homogenates were made in homogenisation buffer (HB) made at 2 × concentration (HB: 10mM Tris- HCl, pH 7.5 containing 0.8 MNaCl, 1 mM EGTA, 1 mM dithiothreitol) containing a 2% sarkosyl detergent and protease and phosphatase inhibitors. Equal amounts of HB buffer were added to the homogenates in a 1:1 ratio to make a final of 1× HB with 1% sarkosyl. The homogenates were then incubated at 37 °C for 30 min and centrifuged (12,000 ×g, 10 min). The supernatant was removed and then ultracentrifuged (100,000 ×g, 10 min, room temperature). The pellet was then washed and sonicated with PBS then re-ultracentrifuged at 100,000 x g for 20 mins at room temperature. The final pellet was resuspended in 50µl of sterile PBS and protein was measured using a BCA assay. To serially passage this material we co-transfected wild type TDP-43 plasmid (as used previously in Smethurst et al., 2016) and 50ug of the insoluble extract to confluent 10cm well plates of HEK293 cells and incubated for 3 days. The cells were then harvested and extracted in 1ml of TS buffer (150 mM Tris HCl, 50 mM NaCl, 5mM EDTA, and 5mM EGTA) containing 1% sarkosyl and protease and phosphatase inhibitors. The samples were sonicated and incubated at 37°C for 30 min. The samples were briefly spun at 16,000 x g for 10 mins and then the supernatant was ultra-centrifuged at 290,000 × g for 20 min at room temperature. The supernatant was used as the sarkosyl soluble (SS) fraction and the pellet was resuspended by sonication in 250ul of sterile PBS then briefly spun at 2000rpm for 5 minutes. A BCA assay was performed on the PBS fraction, and 50ug was co-transfected with wild type TDP-43 to another 10cm well plate of HEKs to serially passage the seed. This process was repeated up to 10 times to increase the yield of phosphorylated TDP-43 protein in the extract which was used as a proxy for the amount of TDP-43 seeds in the extract.

Human iPSC derived MN and AC culture
For all experiments a minimum of 3 different control hiPSC lines were utilized (each in technical triplicate). The hiPSC derived MNs and ACs were cultured and differentiated as previously described (Hall et al., 2017), Briefly, for differentiation, control iPSC-derived ventral spinal neural progenitor cells (NPCs) were propagated in N2B27 medium (DMEM/F12 Glutamax, Neurobasal, L-Glutamine, N2 supplement, non-essential amino acids, B27 supplement, β-mercaptoethanol (all from Life Technologies) and insulin (Sigma) with FGF-2 (Peprotech) for up to 30 days. For terminal differentiation of the NPCs to MNs the cells were plated out onto 0.07% polyethyleneimine (PEI) (Sigma) and Geltrex (Life Technologies) coated 24 well plates into N2B27 media containing compound E (Enzo Life Sciences) to promote cell cycle exit. To produce ventral spinal ACs the NPCs were propagated in FGF-2 for a minimum of 60 days, during which time they had become glial precursor cells (GPCs). FGF-2 withdrawal and co-treatment with 10ng/ml Bone Morphogenetic protein 4 (BMP4) (R&D systems) and 10ng/ml Leukaemia inhibitory factor (LIF) (Sigma) were used to differentiate for a minimum of 3 weeks.

TDP-43 seeding to hiPSC derived MNs and ACs
The hiPSC-MNs were differentiated for a minimum of 3 days and then 5µg of either control spinal cord extract or serially passaged ALS spinal cord extract containing seeds of pathological TDP-43 were either mixed with 4µl of PULSin (Polyplus transfection) reagent in HEPES buffer for 15 mins or with 1.5ul of Lipofectamine 3000 for 5 mins, and then complexes were added to the cells and incubated for 6 hours. After this the complexes were removed, and the media was changed to fresh N2B27 media with compound E. The cells were then fixed at either 3, 7- or 14-days post treatment. For proteasome inhibition, the cells were co-treated with 2µM of MG132 (Sigma) for 6 hours at the same time as transfection of the serially passaged ALS SC.

Spreading and propagation of TDP-43 aggregates in co-culture
To investigate the spreading and propagation of TDP-43 aggregates between MNs and ACs we performed co-culture experiments. For MN to AC propagation, MNs were plated on to 24 well plate coverslips and seeded with serially passaged sALS (spALS) SC extract and MG132 treatment and then ACs were added in a 1:1 ratio the day after the MNs had been washed 3 times with N2B27 media changes. Cells were then fixed in 4% PFA at 3, 7 and 14 days post co-culture. For AC to MN propagation, the ACs were seeded with spALS SC extract and MG132 treatment and washed extensively with media changes. The following day the ACs were dissociated with trypsin and added to MNs in a 1:1 ratio. These co-cultures were then left for 3, 7 and 14 days with regular media changes and fixation with 4% PFA at each of these time points.

Cytotoxicity assay
Toxicity was assayed using the 3-(4,5-dimethylthiazol-2-yl)-2,5-diphenyl-2H-tetrazoliumbromide (MTT) assay which was performed using MTT salt (Sigma) resuspended at 1mg/ml in sterile water. Cells were plated out in 96 well plates at 20,000 cells per well treated with either control or ALS treated cell extracts and then 20µl of the 1 mg/ml MTT solution was added to each well and left to incubate at 37°C for 2–4 h. The media from each well was decanted and replaced with 100µl of dimethyl sulfoxide (DMSO) and agitated until the entire purple formazan product was dissolved and the plate was read at 570 nm.

Absorbance readings were taken as a percentage of the mean value of non-seeded un-treated control cells.

Dot blots
2ul of sample was added to a nitrocellulose membrane and allowed to air dry. The membrane was then blocked in 5% non-fat milk for 30 mins then incubated in primary antibody (polyclonal rabbit anti-TDP-O (1:500, Gift from Dr Yu Run Chen)) for 1 hour in 5% non-fat milk in PBS + 0.01% Tween (PBST) and then washed 3 times for 5 mins each in PBST. The membrane was then incubated with secondary fluorescent IR-Dye 800 at (1:10,000, Abcam) in 5% non-fat milk in PBST for 1 hour and then membrane was washed again 3 time in PBST and developed using the Odyssey Scanner (Li-Cor)

Construction of vector
DNA encoding human TAR DNA-binding protein 43 (TDP 43) was purchased from Eurofins Scientific UK. The codons were optimised for expression in HEK293 cells. The expression vector was prepared using the pTriEX Ek/LIC cloning kit (Novagen, USA). Briefly, the gene was amplified by PCR to incorporate the LIC extensions, gel purified, treated with T4 DNA polymerase, and the resulting fragment was annealed with the pTriEX-4 Ek/LIC vector according to the manufacturers' instructions. The resulting TDP-43-pTriEX Ek/LIC plasmid was transformed into NovaBlue Competent Cells (Novagen). Positive clones were selected on LB agar plates containing ampicillin (100µg/ml) and were analysed for presence of insert by PCR and then sent for DNA sequencing to check the sequence.

Expression of TDP-43-pTriEX EK/Lic into HEK-293F cells
Human embryonic kidney (HEK) -239F cells were grown in Freestyle 293 expression media (Thermofisher) into 2 litres flasks to 1*106 cells/ml and transfected by mixing 1.25µg TDP 43-pTriEX EK/LIC and 1.87 µg of polyethylenimine per ml of cell culture. Cells were incubated on an orbital shaker at 37C, 8% CO2 for 3 days.

Purification, refolding and concentration of TDP-43
The cell pellet was resuspended and lysed into ice cold RIPA Buffer (50mM Tris, 150mM NaCl, 0.5% Sodium Deoxycholate, 0.1% SDS, 1% Triton X100, pH8.8), complete EDTA-free protease inhibitor mixture (Roche). The lysate was centrifuged at 17500xg for 1h, and the supernatant loaded onto a 5ml NiNTA column (GE Healthcare) and eluted in 6M Guanidinium Hydrochloride, 10mM β-mercaptoethanol, 50mM Tris, 1M Imidazole, pH6.8. The sample was then loaded on SDS-PAGE and the band corresponding to the protein was cut and analysed by MALDI-MS. The denatured protein was then refolded by 3 consecutive dialysis of 6 hours at 4°C in PBS, 5mM Reduced Glutathione (GSH), 0.5mM Oxidised Glutathione (GSSG), 0.2M L-Arginine, pH10 followed by dialysis in PBS, 5mM Reduced Glutathione (GSH), 0.5mM Oxidised Glutathione (GSSG), 0.1M L-Arginine, pH10 and finally a dialysis in 20mM Tris, 150mM NaCl, pH10. The TDP-43 oligomers were then concentrated and buffer exchanged with 20 mM Tris Base pH 8.5 using an Ultra-15 Centrifugal Filter Unit with a 100kDa cut off (Amicon).

Characterisation of TDP-43 by SEC-MALS
The protein was loaded onto a Size Exclusion Chromatography Superdex S200 10/30 (GE Healthcare) equilibrated in 20mM Tris, 150mM NaCl, pH10 and eluted using a HPLC

instrument (Agilent) chained with a Dawn HeleosII MALS detector and Optilab dRX refractometer (Wyatt Technology). Molar masses were calculated from the intensity of scattered light at 18 different angles, and SEC elution volume using ASTRA software (Wyatt Technology).

Immunocytochemistry
Cells were gently washed with PBS and fixed for 15 min in 4% PFA. The cells were then washed again in PBS and permeabilised with 0.5% triton X-100 in PBS for 10 min. The coverslips were blocked in 5% BSA for 30 min and stained with mouse monoclonal anti beta-tubulinIII (biolegend), polyclonal goat anti-ChAT (1:100) (Millipore), monoclonal mouse anti-SMI32 (1:1000) (Cambridge Bioscience), monoclonal mouse anti-GFAP (1:200) (Dako), mouse monoclonal anti-Aldh1L1 1:100 (Millipore), monoclonal rabbit anti-TDP-43 (1:500) (Proteintech), monoclonal mouse anti-TDP-43 (1:500) (Proteintech), polyclonal anti-rabbit phospho-TDP-43 (1:500) (Proteintech), polyclonal rabbit anti-complement C3d (1:1000) (Dako) or polyclonal rabbit anti-cleaved caspase 3 (1:400) (Cell Signalling) in 5% BSA for 1 hour at room temperature. Cells were washed in PBS 3 times and incubated with fluorescent secondary anti-rabbit Alexa Fluor 488 (Life Technologies) and anti-mouse or anti-rabbit Alexa-Fluor 568 (Life Technologies) antibodies (1:2000) in 5% BSA for 1 h at room temperature and washed again as before. They were mounted with DAPI Fluoromount G onto slides, allowed to dry and visualised with a Zeiss 710 upright confocal microscope.

Image analysis
All images were analysed using the Volocity software. Nuclear cytoplasmic TDP-43 was calculated using imageJ by separating the TDP-43 and DAPI channel and thresholding and making the DAPI channel binary to use as a template to make images of the nuclear and cytoplasmic TDP-43 only. These images where then measured for pixel intensity and the integrated intensity was used as the final measurement value. These values were added together to make a total value and then percentage of each channel was calculated as the final values. Cell counting was performed using 5 random fields from each coverslip and counted manually using the cell counter facility on the Volocity software.

Statistical analysis
An unpaired two-tailed student's t-test was used when comparing between two individual groups cases to generate a statistical p value. A one-way ANOVA was used when comparing 2 or more groups with a post hoc Tukey test to compare all groups. Any p value below 0.05 was considered to be statistically significant (*$p<0.05$, **$p<0.01$, ***$p< 0.001$).

Compliance with ethical standards
For human iPSC work, informed consent was obtained from all patients and healthy controls in this study. Experimental protocols were all carried out according to approved regulations and guidelines by UCLH's National Hospital for Neurology and Neurosurgery and UCL Queen Square Institute of Neurology Joint Research Ethics Committee (09/ 0272). The human post-mortem spinal cord samples were obtained from the tissue bank NeuroResource, UCL Queen Square Institute of Neurology, London, UK. Samples were donated to the tissue bank with written tissue donor informed consent following ethical review by the NHS NRES Committee London–Central and stored under a Research Sector Licence from the UK Human Tissue Authority (HTA).

Supplementary Figure 1

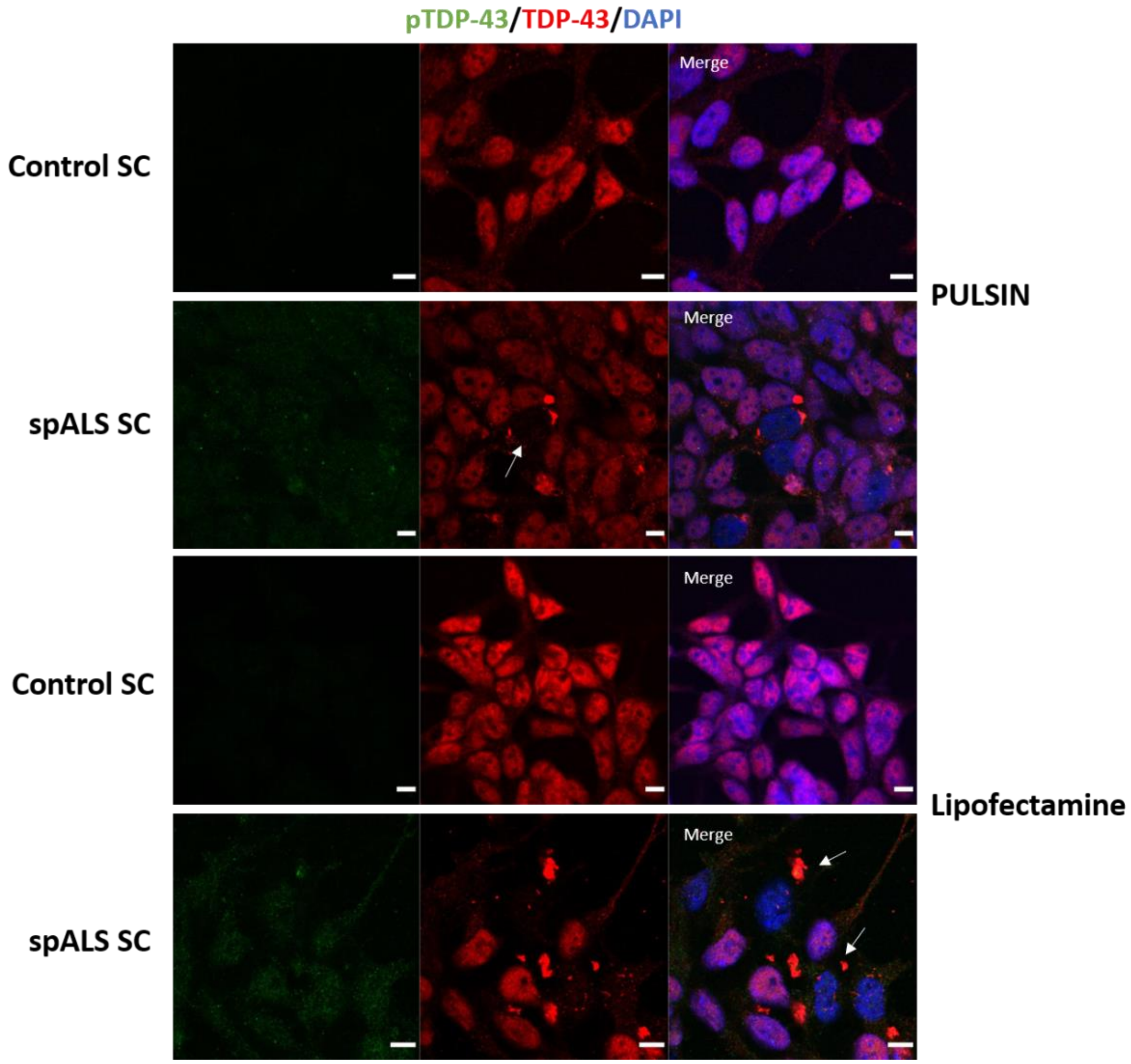

Figure S1. TDP-43 seeded aggregation in non-overexpressing HEK293 cells. Immunostaining demonstrating the seeded aggregation of endogenous TDP-43 3 days after the transfection with either PULSin or Lipofectamine of spALS SC extract that is not attributable to the extraction procedure itself (Control SC). White arrows indicate cells with nuclei cleared of endogenous TDP-43 in the presence of cytoplasmic TDP-43 aggregates. SC = spinal cord, spALS = serially passaged sporadic ALS post-mortem tissue, pTDP-43 = phospho TDP-43 (pS409/410). All scale bars represent 10 µm.

Supplementary Figure 2

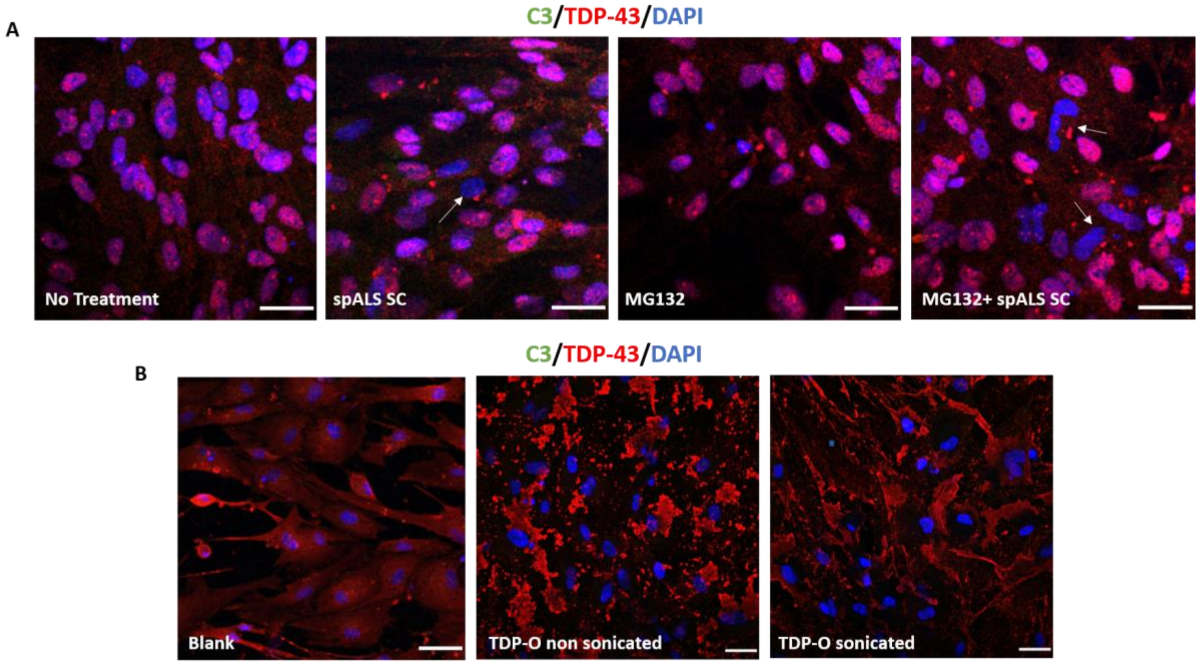

Figure S2. TDP-43 seeded aggregation or toxic oligomers do not cause astrocyte reactivity in hiPSC-ACs. A) Representative images of astrocytes stained / immunolabeled with DAPI (blue), TDP-43 (red) and C3 (green) with no treatment, MG132, spALS SC and MG132 + spALS SC treatments demonstrating lack of C3 positive immunostaining in the presence of TDP-43 aggregates. White arrows indicate cells with nuclei cleared of endogenous TDP-43 in the presence of cytoplasmic TDP-43 aggregates. All scale bars represent 14µm. **B)** Representative immunostaining of ACs treated with non-sonicated or sonicated 500nM TDP-O for 24 hours and stained for DAPI (blue), TDP-43 (red), and C3 (green). SC = spinal cord, spALS = serially passaged sporadic ALS post-mortem tissue. All experiments are n=3 from different control cell lines, error bars = SEM, * = $p<0.05$ ** = $p<0.01$ and *** = $p<0.001$.

Supplementary Figure 3

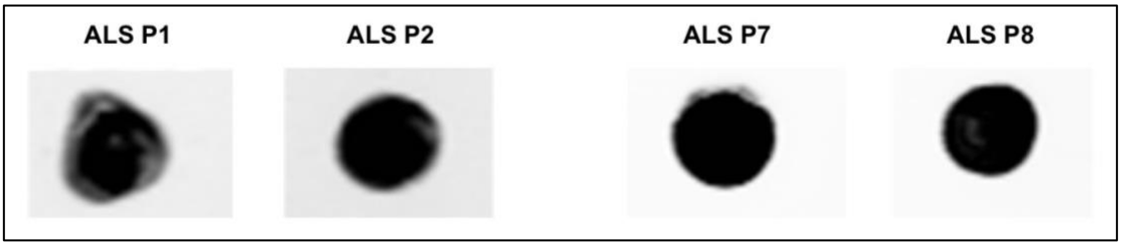

Figure S3. Dot blot with TDP-43 oligomer-specific antibody shows presence of TDP-43 oligomers in multiple serial passages of the ALS spinal cord extract. P1 = passage 1, P2 = passage 2, P7 = Passage 7, P8 = passage 8.
